# Editorial: Modern Chemical Routes for Controlled Synthesis of Bimetallic Nanostructures

**DOI:** 10.3389/fchem.2021.640665

**Published:** 2021-04-06

**Authors:** Stefanos Mourdikoudis, Shinya Maenosono, Catherine Dendrinou-Samara, Jorge Pérez-Juste, M. Rosa Axet

**Affiliations:** ^1^Department of Inorganic Chemistry, University of Chemistry and Technology, Prague, Czechia; ^2^School of Materials Science, Japan Advanced Institute of Science and Technology, Nomi, Japan; ^3^Department of Chemistry, Aristotle University of Thessaloniki, Thessaloniki, Greece; ^4^Departamento de Quimica Fisica, Centro Singular de Investigaciones Biomédicas, Universidade de Vigo, Vigo, Spain; ^5^UPR8241, Laboratoire de Chimie de Coordination, Université de Toulouse, UPS, INPT, CNRS, Toulouse, France

**Keywords:** bottom-up, colloidal synthesis, catalysis, antimicrobial, LSPR

The performance of a variety of nanostructure types in a range of domains necessitates a careful control of their size, shape, crystal structure, and composition. In the case of bimetallic nanomaterials, another parameter that has to be taken into account is the way with which two given metals can be combined. Under certain circumstances, this configuration can be an alloy, a core-shell structure or a heterostructure. Often, the presence of synergistic effects between both metals can endow improved properties to the final bimetallic nanostructures, compared to their single-metal components. A convenient way to achieve bimetallic nanostructures with well-tuned characteristics is the use of colloidal chemical bottom up synthetic pathways. In this context, parameters that can be tailored to obtain the desired nanomaterials concern the amount and kind of capping agents, reductants, solvents, and precursors together with the synthesis temperature. Consequently, bimetallic particles can be synthesized in an optimized way, with improved physical properties, thus being promising for application in different fields, such as catalysis/electrocatalysis, biomedicine, environment, and energy. For example in chemical catalytic and electrocatalytic reactions bimetallic nanostructures can be applied either as unsupported particles or integrated/embedded in proper matrices, such as reduced graphene oxide. Biomedical uses include antibacterial function, anticancer treatment, biological imaging, and drug delivery. Environmental applications concern CO oxidation and hydrodechlorination, among several others.

Considering the fact that the chemical synthesis of bimetallic nanostructures composes a timely topic of research nowadays, we invited experts from around the globe so as to form a relevant Research Topic. As a result of our efforts, the collection of published papers consists of three research papers, two Review papers and one Mini Review paper.

In particular, Bhugra et al. reported the production of Ni_1−*x*_Fe_x_ nanofibers through means of electrospinning. High saturation magnetization was observed, whereas the coercivity was higher than that of the corresponding bulk material. More insights on the magnetic behavior of the fibers, probably related to a surface composition difference, were delivered by the authors. Potter et al. presented a unique synthetic design strategy to form multi-dopant solid-acid catalyst (MgSiAPO-34). This was compared with the performance of its mono-dopant analog, SiAPO-34 for the ethanol to ethylene dehydration reaction. The improved catalytic activity of the multi-dopant catalyst was explained. Antonoglou et al. published the polyol-mediated synthesis of brass (CuZn) nanoparticles under different heating conditions (autoclave system, range of temperatures, conventional or microwave heating). Distinct hydrophilic phases with tuned structure and compositions (from Cu-rich to Zn-rich) were isolated.

Bhol et al. reviewed the synthesis pathways of transition metal bimetallic anisotropic nanostructures for use in catalysis. The authors provided insights on how even subtle changes in the reaction conditions can modify the growth pattern, thus altering the final anisotropic nanostructures, affecting also their catalytic performance. The antimicrobial performance of unsupported and supported bimetallic nanostructures was the topic which Arora et al. decided to overview. Examples of supports described were graphene, zeolites, clays, fibers, and polymers. The association between preparation routes, properties and antimicrobial activity was analyzed. Furthermore, Min and Wang highlighted in their mini review the recent advances in manipulating the localized surface plasmon resonance (LSPR) properties of bimetallic nanostructures, together with their applications in sensing. They discuss how the control of elemental proportion and spatial arrangement of the two metals, as well as the adjustment of their size and shape influence the resulting LSPR features. As a consequence, the tuning of the LSPR signal will then offer an ameliorated sensitivity and reproducibility of the sensing capabilities of those bimetallic systems.

We believe that the publication of this Research Topic comes in a timely manner. The balance between research and review papers will help readers to see the topic from different angles. The interest for the Research Topic is expected to be multidisciplinary, coming from chemists, materials scientists, biologists and engineers. A better comprehension on how spherical and anisotropic bimetallic nanostructures can be wet-chemically produced in a controlled way will motivate scientists to improve protocols from the literature and design their own ones, targeting to acquire nanoparticles with finely-tuned features. Especially when aiming “real-life” applications, it is of utmost importance to ensure optimized, highly reproducible laboratory-scale synthetic protocols, which display an increased possibility for a successful scale-up to real-world, commercial/industrial level. In this manner, the synergistic effects thanks to the presence of two different metals will be better exploited and the prepared nanomaterials will become more promising to be used in a variety of domains. We would like to thank all the authors and reviewers for their contributions that led to the completion of this Research Topic. In addition, the editorial staff and the production team of Frontiers are acknowledged for their help.

## Author Contributions

SMo designed this Research Topic from the beginning, invited the co-editors and possible authors and reviewers, and handled some of the submitted manuscripts. SMa, CD-S, JP-J, and MA invited the authors and reviewers and handled some of the submitted manuscripts. All authors contributed to the article and approved the submitted version.

## Conflict of Interest

The authors declare that the research was conducted in the absence of any commercial or financial relationships that could be construed as a potential conflict of interest.

